# A High Spatial Resolution Depth Sensing Method Based on Binocular Structured Light

**DOI:** 10.3390/s17040805

**Published:** 2017-04-08

**Authors:** Huimin Yao, Chenyang Ge, Jianru Xue, Nanning Zheng

**Affiliations:** 1 The Institute of Artificial Intelligence and Robotics, Xi’an Jiaotong University, Xi’an 710049, China; huimin.yao@stu.xjtu.edu.cn (H.Y.); jrxue@mail.xjtu.edu.cn (J.X.); nnzheng@xjtu.edu.cn (N.Z.); 2 The National Engineering Laboratory for Visual Information Processing and Applications, Xi’an Jiaotong University, Xi’an 710049, China; 3 Shaanxi Provincial Key Laboratory of Digital Technology and Intelligent Systems, Xi’an 710049, China

**Keywords:** depth sensing, binocular structured light, spatial resolution, Kinect, speckle pattern

## Abstract

Depth information has been used in many fields because of its low cost and easy availability, since the Microsoft Kinect was released. However, the Kinect and Kinect-like RGB-D sensors show limited performance in certain applications and place high demands on accuracy and robustness of depth information. In this paper, we propose a depth sensing system that contains a laser projector similar to that used in the Kinect, and two infrared cameras located on both sides of the laser projector, to obtain higher spatial resolution depth information. We apply the block-matching algorithm to estimate the disparity. To improve the spatial resolution, we reduce the size of matching blocks, but smaller matching blocks generate lower matching precision. To address this problem, we combine two matching modes (binocular mode and monocular mode) in the disparity estimation process. Experimental results show that our method can obtain higher spatial resolution depth without loss of the quality of the range image, compared with the Kinect. Furthermore, our algorithm is implemented on a low-cost hardware platform, and the system can support the resolution of 1280 × 960, and up to a speed of 60 frames per second, for depth image sequences.

## 1. Introduction

At present, human computer interaction based on three-dimensional (3D) depth information has become highly attractive in the areas of image processing and computer vision, which further promotes the development of 3D depth acquisition technology. In addition, the recently developed 3D depth sensors, such as the Microsoft Kinect (Microsoft Corporation, Redmond, Washington, DC, USA) [1], have been applied in more fields, such as gesture recognition [2,3,4,5], intelligent driving [6,7], surveillance [8], 3D reconstruction [9,10], and so on. 3D depth acquisition technology measures the distance information between objects and a depth sensor. It represents a non-contact, non-destructive measurement technology.

The fully studied methods for acquiring depth information can be classified into two categories: passive methods and active methods.

Binocular stereo vision is an active research topic in passive methods. Some authors [11,12] have implemented an entire stereo vision process on a hardware platform accompanied by progress of the algorithms and field programmable gate array (FPGA) technology. However, the considerable computational expense still limits its industrialization, as some algorithms have to be realized on a custom-build FPGA. One the other hand, stereo vision still does not have a good method to acquire the dense disparity map for a large textureless area, such as a white wall. Therefore, no related electronic products have yet been released.

Time-of-Flight (ToF) and structured light [13,14] is mainly used in the active depth sensing method. The ToF technology acquires the distance information based on measuring the flight time along the light path. Samsung released the first complementary metal oxide semiconductor (CMOS)-based ToF sensor in 2012, where synchronized depth (480 × 360) and RGB images (1920 × 720) can be obtained by a single image sensor [15]. The Kinect 2 (Kinect for XBox One) issued in 2013 by Microsoft is also based on the ToF principle [16]. However, in terms of miniaturization and integration, the advantages of technology based on structured light are not ignored. In addition to the Microsoft Kinect, other companies have also researched and released their own depth-sensing cameras, or intend to integrate them into their electronic products. For example, in 2013 Apple purchased Primesense and claim an important invention patent: “Depth Perception Device and System” [17], and intends to employ it as an input device for human–machine interfaces for their product. In early 2014, Intel announced a three-dimensional depth imaging device, the “RealSense 3D Camera,” and the products RealSense R200 and SR300 have been on the market since 2016. In February 2014, Google announced a new project “Tango,” which intends to make a smartphone with 3D visual recognition capabilities. In 2015, the Microsoft holographic visor “Hololens” appeared on the market.

However, the depth-sensing systems based on structured light are not mature when compared to two-dimensional (2D) imaging systems. The resolution and accuracy of the depth image are lower, and the performance is not very reliable in the use of moving objects or dynamic scenes. In [18,19], the authors combine color images to restore the corresponding depth image. The methods can improve the image quality and reduce the noise of the acquired depth map, but the improvement of the measurement precision is limited.

Moreover, the hardware implementation of depth sensors based on structured light remains a black box, to a large extent, in the literature. In [20], we have proposed a full very large scale integration (VLSI) implementation method to obtain a high-resolution and high-accuracy depth map based on randomized speckle patterns. Then it was found that the spatial resolution of the depth map has the potential to be further improved. Thus, in this paper, we add an infrared camera at the right of the laser projector and employ the local binocular stereo matching algorithm to improve the performance of the depth map.

Several binocular structured light approaches have been proposed for acquiring a disparity map. Different projected patterns and disparity estimation methods are employed. Ma et al. combined a color-coded pattern based on vertical stripes and the semi-global stereo matching (SGM) algorithm to obtain a dense facial disparity map [21]. An et al. provided the comparative analysis of various structured lighting techniques with a view for facial reconstruction [22]. Nguyen et al. employed the dot-coded pattern to enhance textures on plants, and five pairs of RGB cameras to reconstruct the whole plants [23]. In this system, stereo block matching is applied to calculate the matching results. The gray or color-coded patterns mentioned above are sensible to ambient light and the surface color of objects. Yang et al. used the light stripes and corresponding decoding method to measure the 3D surfaces [24]. However, this method is not suitable for moving objects. The experimental setup proposed by Dekiff et al. also used the speckle pattern and digital image correlation [25]. There is a triangulation angle of about 30° between the two cameras. It is a short-range measurement system. The measuring distance is under 1 m and the size of the measuring field is approximately 24 cm × 18 cm. The Intel RealSense Camera SR300 includes two infrared cameras and has similar performance.

Our key contribution in this paper, beyond [20], is that an infrared camera is added at the right of the laser projector, then the binocular matching mode, performed between the captured left and right patterns, is employed to improve the spatial resolution in the X-Y direction. The mismatching and occlusion is unavoidable in the binocular matching process, so the monocular matching mode, performed between the left and the reference patterns, is also employed to revise the matching results. Finally, the complete system is implemented and verified on a low-cost hardware platform with a laser projector and two-camera setup.

The rest of this paper is organized as follows: In Section 2, the basic principles mentioned in our depth-sensing method are briefly introduced. The acquisition steps of the depth map from projected speckle patterns are described in Section 3. Section 4 gives the full-pipeline architecture of our proposed method. Section 5 discusses the utility of our method and presents some simulation results. The paper ends with some conclusions in Section 6.

## 2. Related Ranging Principles

In [20], a consistency enhancement algorithm is proposed to make the intensity of the pattern formed at different distances as consistent as possible so as to ensure the matching accuracy in the disparity estimation process. However, the surface material of the objects also affects the projected pattern, so we have to select a larger block, compared with the smallest size in theory, to perform the block-matching step. Therefore, we add an infrared camera at the right of the laser projector in order to capture a pattern that is nearly consistent with the captured left pattern. Then the smallest image block can be employed in the binocular matching mode performed between the captured left and right patterns so as to improve the spatial resolution of the depth map.

**Figure 1:**
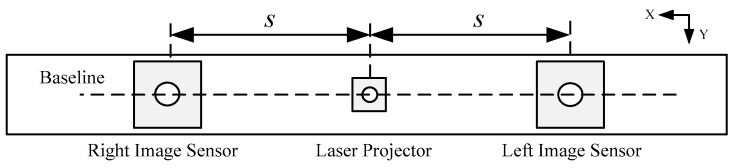
Front view of the experiment platform.

Figure 1 shows the position relationship between the projector and cameras. One camera is added to the right of the laser projector compared with traditional depth-sensing method. The experimental platform contains a projector which projects a speckle pattern similar to that used in the Kinect and two cameras with the same performance parameters. The projector is between the two cameras. The three units are located in the same straight line and the optical axes of each are parallel. In our depth-sensing method, the triangulation principle and digital image correlation (DIC) are related. The triangulation principle is to transform the disparity to distance information through the geometrical relationship. Generally, the digital image correlation (DIC) is performed to identify the corresponding points in the individual images.

### 2.1. Triangulation Principle

**Figure 2:**
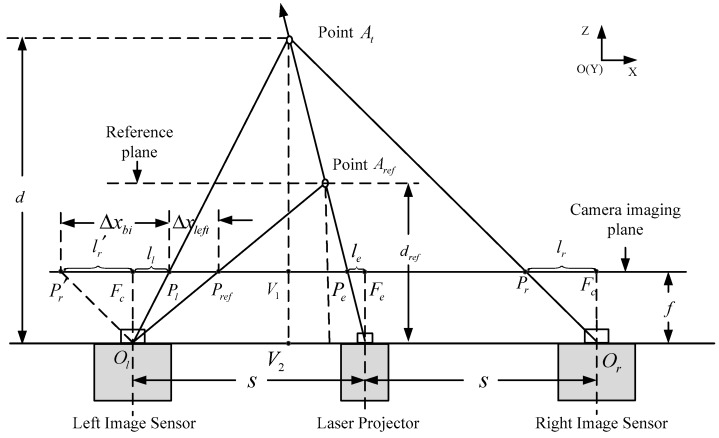
Schematic diagram of the triangulation principle.

In the binocular stereo system, the problem of occlusion is unavoidable. Thus, we combine the monocular ranging method to compensate for the disparity of the occluded areas. In this section, the ranging principle and the transformation relationship of the two displacement vectors from two modes are introduced. The ranging method is based on the triangulation principle. As shown in Figure 2, point $A_{t}$ is an arbitrary point in the 3D space marked by the speckle pattern. The two points, $P_{l}$ and $P_{r}$, respectively, are the projection of $A_{t}$ on the left and right camera imaging planes, so they are the corresponding image points of $A_{t}$ in the left and right two images. According to photogrammetry, if we obtain the displacement of the test point $A_{t}$ in two images, we can obtain the vertical distance from point $A_{t}$ to the ranging sensor. The two cameras are located as shown in Figure 1, and their coordinates only differ as per the translation distance in the X direction. Thus, the image planes of the two cameras are on the same plane, and the epipolar lines are aligned with the scanline of the image. Therefore, the displacement Δy in the Y direction can be ignored, we only require an estimate of the displacement Δx in the X direction between the projection point of $A_{t}$ on the left and right camera imaging planes. We assume that the optical focal length of the image sensor is f, μ is the physical pixel-pitch, and s is the baseline distance between the laser projector and image sensor. According to the relationship between similar triangles, we can obtain the proportional relationship $\frac{d-f}{d}=\frac{2s-l_{l}-l_{r}}{d}$, where the parameter d is the distance between the sensor and the point $A_{t}$. Then the depth value of point $A_{t}$ can be calculated as

(1) $$d=\frac{2sf}{l_{l}+l_{r}}=\frac{2sf}{\mu \Delta x_{bi}}$$

where $\Delta x_{bi}$ represents the displacement of the corresponding image points $P_{l}$, $P_{r}$, respectively, on the left and right images.

Next, the depth sensing technology with only the left-side camera is discussed. In the process, we need to acquire a reference image from the left camera at a known distance. As shown in Figure 2, the point $A_{r}$ is the position of point $A_{t}$ at the reference distance. According to the relationship between similar triangles, we can obtain the proportional relationship $\frac{d-f}{d}=\frac{s-l_{l}-l_{e}}{s}$ and $\frac{d_{ref}-f}{d_{ref}}=\frac{s-l_{l}-l_{e}-\mu \Delta x_{left}}{s}$. Then the depth value of point $A_{t}$ can be calculated as

(2) $$d=\frac{1}{-\frac{\mu }{sf}\Delta x_{left}+\frac{1}{d_{ref}}}$$

where $\Delta x_{left}$ represents the displacement of the corresponding image points $P_{l}$, $P_{ref}$, respectively, on the left and its reference images.

From Equations (1) and (2), we can observe that the inverse of distance d follows a linear relationship with the displacement $\Delta x_{bi}$ or $\Delta x_{left}$; thus, there is a simple linear relationship between the two disparities. The point $A_{t}$ can be located in front of, or behind, the reference point $A_{ref}$, so the value of displacement $\Delta x_{left}$ can be positive or negative. In this paper, we set a direction for the displacement. The projection point $P_{l}$ is assigned as the end point of the vector, and the projection point $P_{r}$ or $P_{ref}$ as the start point of the vector. Then we can get $\vec{\Delta x_{left}}=\vec{P_{ref}P_{l}}=-\Delta x_{left}$ and $\vec{\Delta x_{bi}}=\vec{P_{r}^{\prime }\;P_{l}}=\Delta x_{bi}$. Combining Equations (1) and (2), we can obtain the relationship of the two displacement as follows:

(3) $$\vec{\Delta x_{bi}}=2\vec{\Delta x_{left}}+\frac{2sf}{\mu d_{ref}}$$

### 2.2. Digital Image Correlation

The digital image correlation (DIC) algorithm was developed at the end of last century and has been widely used to analyze deformation and identify the corresponding points between different images [25,26]. Its principle is to match subsets from different digital images by an appropriate similarity calculation method. The process is to ensure the parameters in the displacement shape function through a correlation function. The displacement mentioned in the Section 2.1 is contained in these parameters.

Assume that the coordinate of point $P_{l}$ in the first image (left speckle image) is $\left(x_{0},y_{0}\right)$. In order to determine the corresponding point $\left(x_{0}^{\prime },y_{0}^{\prime }\right)$ in second image (right or reference speckle image), a square subset around the central point $\left(x_{0},y_{0}\right)$ is extracted. Each point in the subset is represented by (x,y). If the represented subset’s surface is perpendicular to the optical axis, it only brings in rigid displacement; the mapping position $\left(x^{\prime },y^{\prime }\right)$ in the second image of each point in this subset is calculated by:

(4) $$x^{\prime }=x+u$$

where u is the displacement of the subset center point in the X direction (the displacement in the Y direction can be ignored in our paper), while, in most situations, the speckle pattern projected on the tested object may be deformed. Thus we introduce the first-order displacement shape function to approximate the mapping position

(5) $$x^{\prime }=x+u+u_{x}\left(x-x_{0}\right)+u_{y}\left(y-y_{0}\right)$$

where the parameters $u_{x}$, $u_{y}$ are the components of the first-order displacement gradient. Lu and Cary [27] propose a DIC algorithm using a second-order displacement shape function for large deformations, but it has twelve parameters which increases the computational difficulty and complexity. Therefore, the displacement is estimated according to Equation (5). The block-matching algorithm is employed to estimate the displacement u in Equations (4) and (5), which is also $\Delta x_{bi}$ or $\Delta x_{left}$ in Section 2.1. In the implementation process, first we assign different initial values for parameters $u_{x}$ and $u_{y}$, and then extract the different subsets with the size of m×m from the second image. Finally, Equation (6) is employed as similarity criterion to find the optimal matching subsets:

(6) $$C\left(u,u_{x},\;u_{y}\right)=\frac{\sum \text{​}\sum \text{​}f\left(x,y\right)g\left(x^{\prime },y^{\prime }\right)}{\sqrt{\sum \text{​}\sum \text{​}f\left(x,y\right)f\left(x,y\right)}\sqrt{\sum \text{​}\sum \text{​}g\left(x^{\prime },y^{\prime }\right)g\left(x^{\prime },y^{\prime }\right)}},$$

where the f(x,y), $g\left(x^{\prime },y^{\prime }\right)$ are, respectively, the discrete gray value of the first and second patterns.

The digital image correlation (DIC) algorithm is a local stereo matching algorithm. In addition to this algorithm, there are other stereo matching algorithms, such as semi-global block matching [28], belief propagation, graph cuts [29], and so on. However, these algorithms are not adaptable to our system. The reasons are as follows. Firstly, in the structured light ranging, the projected pattern is coded following a certain principle. The decoding method that is the ranging algorithm is designed according to the coding method. In this paper, the speckle pattern is binary, and composed of randomly distributed isolated speckles. Every image block extracted from the pattern is unique, so the block-matching algorithm is enough to decode the pattern. Secondly, other stereo matching algorithms are very complex and utilized to optimize the depth map, such as the problems of texturelessness and occlusion. In our paper, the infrared camera only can capture the projected image itself without additional information from the surrounding environment, such as the color or gray information. The edge information and other features of objects are very difficult to calculate from the captured image, so the optimization of these stereo-matching algorithms for our result is limited. On the other hand, the scene has been marked by the projected spots, so there is no textureless area, and the occlusion area that exists in binocular matching can be corrected by the monocular matching results. Thirdly, our system is implemented and verified on a hardware platform. An iterative algorithm is always used in the other stereo matching algorithms to find the optimal solution of the established energy function while the iterations are different for every pixel or image, so the hardware costs are uncertain, which does not conform to the hardware design principle. Finally, considering the trade-off between accuracy and hardware costs, we employ the block-matching algorithm in our system design.

## 3. The Depth-Sensing Method from Two Infrared Cameras Based on Structured Light

**Figure 3:**
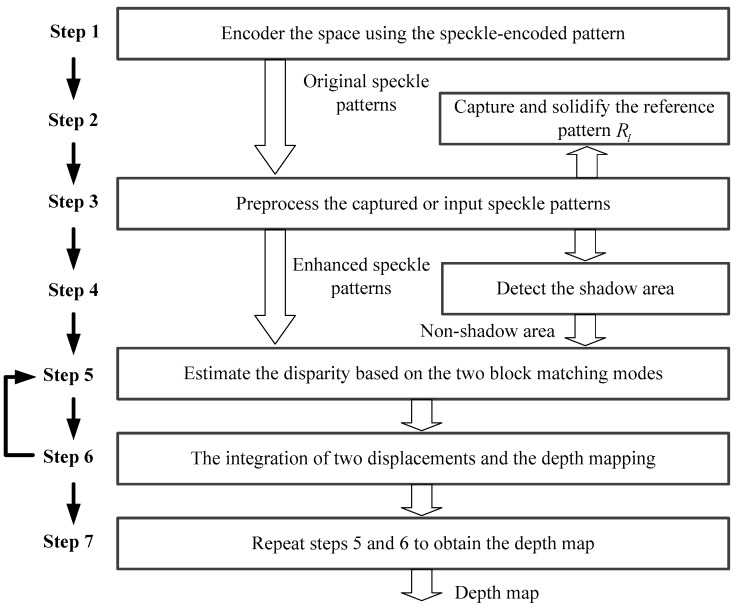
Flow diagram of depth-sensing method.

Figure 3 shows the flowchart of our method. The method can be divided into seven steps and the details are described as follows:

**Step 1: Encode the space using the speckle-encoded pattern.**

Based on the active mode, the projector projects the speckle-encoded pattern to encode or mark the space or object. The pattern is fixed and composed by the randomly distributed spots that are formed by interference of coherent laser beams. The basic design principle of projected patterns is that the pattern in any sub-window is unique and fully identified.

**Step 2: Capture and solidify the reference pattern** $R_{l}$ **from the left camera.**

As shown in Figure 1, the two identical cameras are symmetrically arrayed on the both sides of the pattern projector and the optical axes of the cameras and projector are parallel with each other. The narrow band-pass filters are pasted on the lens of two cameras so the infrared light within a scope of certain wavelengths, which are adopted by the projector, can only be captured. This design mainly eliminates the disturbance of other wavelengths of light or sources and obtains a clear and stable encoded pattern projected by the projector. 

Before working, we need to capture and solidify the left-side reference pattern for disparity estimation. The reference pattern is generated by capturing the speckle pattern projected on the standard plane perpendicular to the optical axis (Z-axis) of the laser projector and cameras. The vertical distance $d_{ref}$ between the standard plane and the depth sensor is known. The selection must ensure that a majority part, or the whole speckle pattern, can be projected onto the standard plane and can also occupy the entire field of view of the left image sensor. Then the reference speckle pattern is processed by the speckle pattern preprocessing module described in step 3, and the fixed image is stored in memory. In the normal processing stage, the system only needs to read the reference speckle pattern from memory for the disparity estimation of the captured speckle pattern image.

**Step 3: Preprocess the captured or input speckle patterns** $I_{l}$**,** $I_{r}$ **from the two cameras.**

For the original speckle patterns taken directly from the cameras, the intensity and the size of the spots formed with laser interference decrease with the increment of the projection distance and may be uneven in the whole image. Hence, a consistency enhancement algorithm proposed in [20] is used to enhance the input speckle patterns to make it more discriminative so as to improve the matching accuracy. The algorithm combines grayscale transformation and histogram equalization, is suitable for hardware implementation, and can be described as

(7) $$f^{*}\left(x,y\right)=\{\begin{array}{cc} \beta \times \left(f\left(x,y\right)-f\left(x,y\right)\right), & \mathrm{if}\;\;\;f\left(x,y\right)\geq \bar{f}\left(x,y\right)\\ 0, & \mathrm{if}\;\;\;f\left(x,y\right)\leq \bar{f}\left(x,y\right) \end{array}$$

where $\bar{f}\left(x,y\right)$ is the average gray value of the subset with the center pixel (x,y) and $\beta =gray_{ref}/\bar{f}\left(x,y\right)$ is a scale factor. 

**Step 4: Detect the shadow area of the enhanced patterns** $I_{l}$**,** $I_{r}$**.**

**Figure 4:**
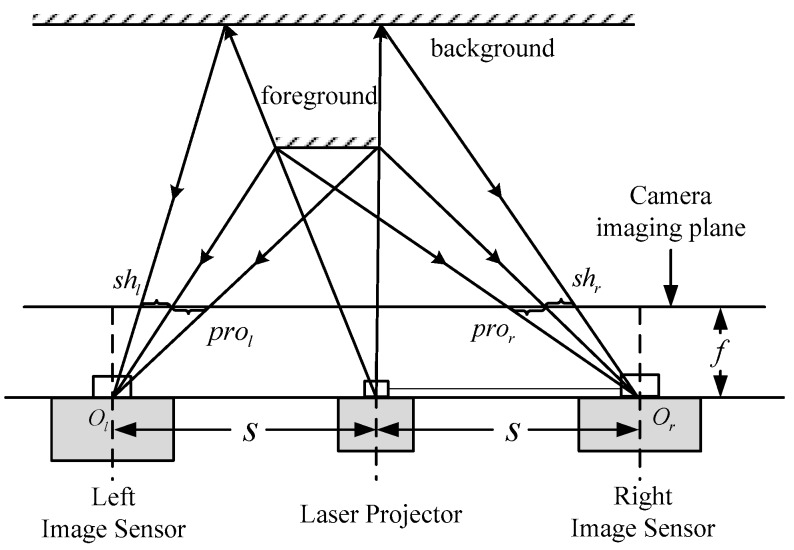
Schematic diagram of the principle of shadow formation.

As shown in Figure 4, because of the distance difference between the foreground and background, a blank area or no projection area is formed, that is, the shadow area. The subset in the shadow area is unable to use Equation (6) to calculate the correlation coefficient. This will affect the next disparity estimation step. Hence, in the preprocessing module for the image $I_{l}$ and $I_{r}$, the shadow area is detected and marked. The disparity estimation will not be performed in the marked area. The shadow area detecting method used to detect the number of pixels of speckle spots within a certain size of the subset. If the number is less than a threshold, the center pixel of the subset is in the shadow area, otherwise the pixel is in the projection area. 

For the left camera, the shadow area $sh_{l}$ is located at the left side of the projection $pro_{l}$ of the foreground while, for the right camera, the shadow $sh_{r}$ is at the right side of $pro_{r}$. It is clear that the edge of the shadow area is correlated with the edge of the foreground. This characteristic can be used to segment the object located in the foreground. 

**Figure 5:**
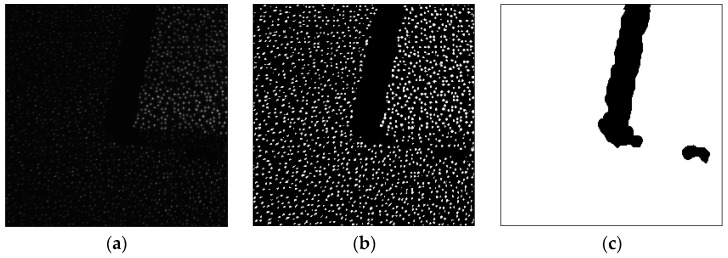
The speckle pattern and the results after preprocessing: (**a**) the speckle pattern; (**b**) the consistency enhancement result; and (**c**) the shadow detection result.

The results after preprocessing for a captured speckle pattern are shown in Figure 5. It is obviously that, in Figure 5b, after consistency enhancement, the intensity of spots in whole speckle pattern is more consistent. The captured speckle pattern is from left camera, thus in Figure 5c, the shadow of the object is at the left side of the projection.

**Step 5: Estimate the disparity based on the two block matching modes.**

In binocular matching, occlusion is unavoidable. In the matching between left and right patterns, there are mismatching areas caused by occlusion. In order to solve this problem and improve the matching accuracy, we employ two matching modes in our paper. One is to estimate the disparity between the left and right pattern, called the binocular mode, another is between the left pattern and its reference pattern fixed in memory, called the monocular mode.

**Figure 6:**
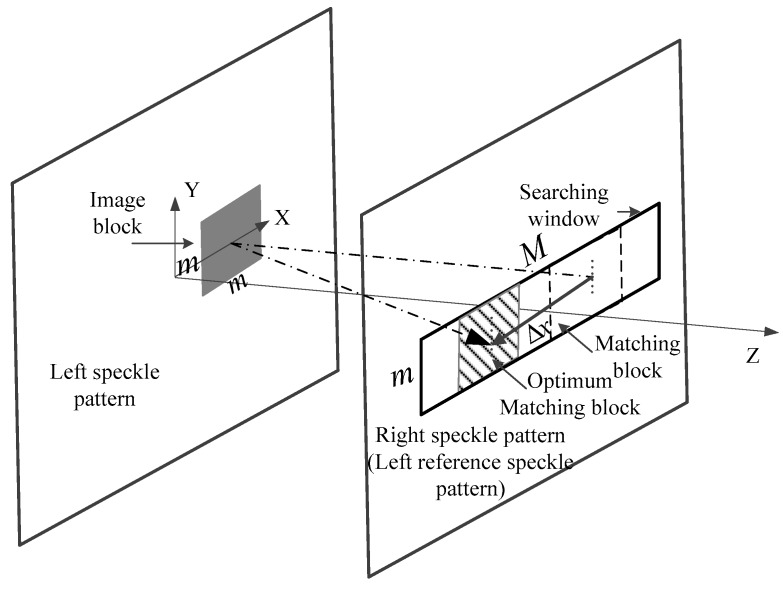
Schematic diagram of the block-matching-based disparity estimation.

For the monocular mode, Equation (4) is employed to estimate the displacement. As shown in Figure 6, we extract a square image block (or subset) of size m×m from the left speckle pattern. Due to the position of the image sensors and the laser projector, the block matching algorithm is confined along the X-axis only. Thus, a search window of size m×M from the left reference speckle pattern is extracted, and the central pixel of the image block and the searching window share the same Y coordinates. The m parameters are odd numbers, and m≪M. Then the full-search block-matching method and the correlation Equation (6) are used to find the optimum matching block and estimate the displacement $\Delta x_{left}$ of the center pixel of the image block. Finally, we use Equation (3) to convert the displacement $\Delta x_{left}$ to $\Delta x_{bi}^{\prime }$.

For the binocular mode, the baseline length is twice that of the monocular mode; therefore, the deformation is larger and needs to be considered. In the matching process, Equation (5) is employed to estimate the displacement. The parameter $u_{x}$ is set as 0, and $u_{y}$ is set as –3/8, 0, 3/8, respectively. We round the calculated the pixel position to extract a square block from search window. The remaining process is the same as the monocular mode and then we acquire the displacement $\Delta x_{bi}$ for the center pixel of the image block.

**Step 6: The integration of two displacements and the depth mapping.**

**Figure 7:**
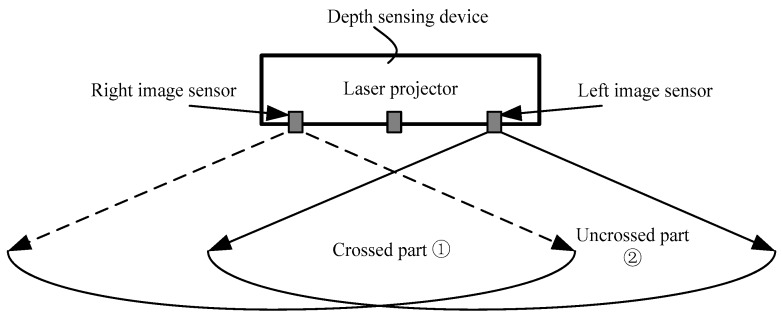
The field of view (FoV) of the output depth image about the two working modes.

As shown in Figure 7, the field of view (FoV) of the left image sensor can be divided into two parts. One is the field of the crossed part, the other is the uncrossed part. For the pixel in the crossed part, if $\left|\Delta x_{bi}^{\prime }-\Delta x_{bi}\right|<th$, then $\Delta x_{bi}$ is selected as the final displacement Δx, otherwise we compare the maximum correlation coefficients of the two matching modes and the larger one is selected as the final displacement Δx. For the pixel in the uncrossed part, we cannot obtain the displacement $\Delta x_{bi}$, therefore, the $\Delta x_{bi}^{\prime }$ is selected as the final displacement Δx.

Based on the obtained the final displacement Δx, we can calculate the distance information for the target object by triangulation according to Equation (1).

**Step 7: Obtain the depth map.**

Move the center pixel of the image block to the next neighboring pixel in the same row, and repeat steps 5 and 6 to obtain distance information for the neighboring pixel. Then the whole depth image can be obtained on the pixel-by-pixel and line-by-line basis.

## 4. Hardware Architecture and Implementation

**Figure 8:**
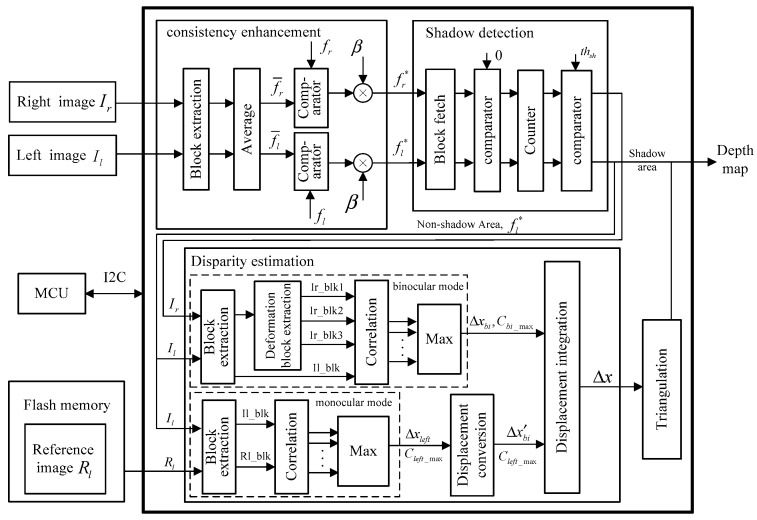
Architecture of the depth sensing method.

In this paper, our method is implemented on a hardware platform. Figure 8 shows the architecture of our depth-sensing method. There are three main modules in the FPGA, i.e., consistency enhancement, shadow detection, and disparity estimation, and two external modules, the microcontroller unit (MCU) and flash memory. On the one hand, the MCU controls the reading and writing of the flash memory. Before processing, the reference image $R_{l}$ needs to be solidified in flash memory in advance, so the MCU will send a writing signal to control the flash memory through the inter-integrated circuit (I2C) bus. In the processing stage, the FPGA reads the reference data from flash memory; thus, the writing signal needs to be changed as a reading signal. On the other hand, there are some thresholds in our method, such as the $gray_{ref}$ in the consistency enhancement and the threshold th in the displacements integration step. The MCU help us to adjust these thresholds outside the FPGA.

**Figure 9:**
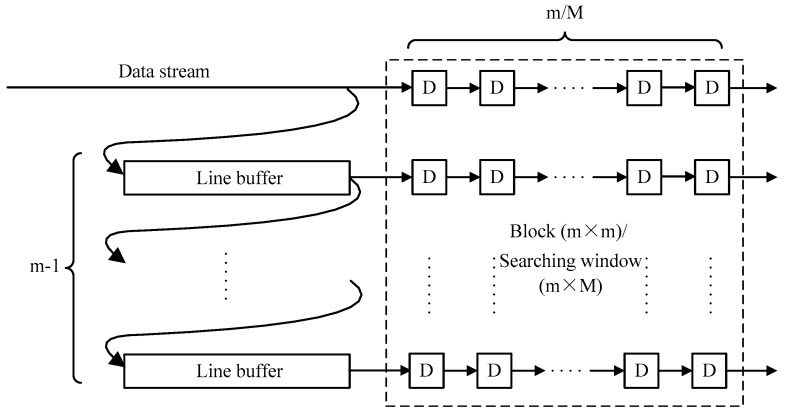
Block extraction submodule.

The pipeline framework is the heart of the hardware implementation. For example, the input image is scanned from the upper-left pixel to the lower-right pixel to generate a fixed data stream. However, every pixel is processed with the surrounding pixels in steps 3, 4 and 5. Then the block extraction submodule is essential in the three main modules. As show in Figure 9, the m−1 line buffers and m×m D-trigger are used in this submodule to extract a m×m image block. The length of the line buffer is equal to the image width and each D-trigger represents a clock cycle delay. The extraction of the searching window also uses the same architecture, except the length of the D-trigger is changed to M.

In consistency enhancement module, after the block extraction module, there is an average submodule to calculate the average gray value of the extracted image block. Then comparator and multiplier are employed to enhance the central pixel according to Equation (7). In the shadow detection module, the comparator and counter are employed for the extracted image block to detect whether the central pixel is in the shadow area.

**Figure 10:**
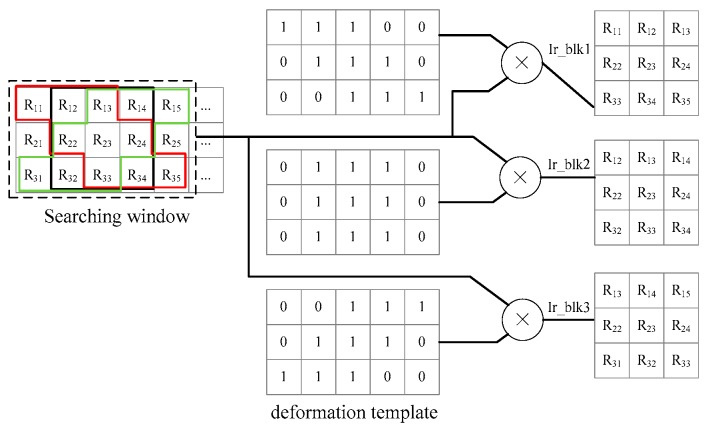
Deformation block extraction submodule.

In disparity estimation, after the extraction of the image block and the searching window, the M−m+1 matching blocks in the searching window and image block are fed into the correlation submodule to calculate the correlation coefficients according to Equation (6). Then, the max submodule selects the max coefficient $C_{max}$ and acquires the corresponding displacement. However, there is a slight difference in the binocular mode because the deformation is considered in our paper. The matching block extraction contains an extra submodule, i.e., the deformation block extraction submodule, and an example for this submodule is given in Figure 10 by m=3 and $u_{x}=0$, $u_{y}=\pm 1,\;0$. For every central pixel in the searching window, we extract three matching blocks marked in red, black, and green. The deformation templates have been stored in advanced. Then a 3×5 block with central pixel $R_{23}$ is multiplied with the three templates. If the pixel in the template is one, the corresponding pixel in the block will be retained to form the matching block.

For the last two submodules, displacement conversion and triangulation, we establish two lookup tables (LUTs), which store the calculating results in ROM, according to Equations (1) and (3) in Section 2.1, respectively. Every input displacement corresponds to a register address, and the result is read from the corresponding address.

## 5. Experimental Results and Discussion

**Figure 11:**
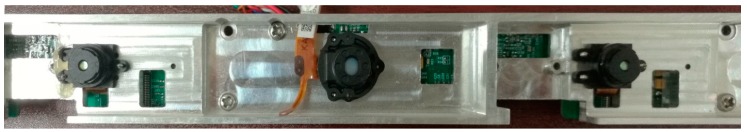
The hardware test platform.

We designed an FPGA hardware platform, as shown in Figure 11, to verify the depth sensing algorithm proposed in this paper. On the platform, we use the near-infrared laser projector (emitting laser speckle pattern), similar to that used in Kinect and two identical IR image sensors (receiving the laser speckle pattern, with output resolution supporting 1280 × 960 at 60 Hz), which are fixed on an aluminum plate. Our proposed depth-sensing method is verified on an Altera FPGA (type: EP4CE115F23C8N, Intel Corporation, Santa Clara, CA, USA).

### 5.1. The Validation of the Transforming Relationship between Two Displacements

**Figure 12:**
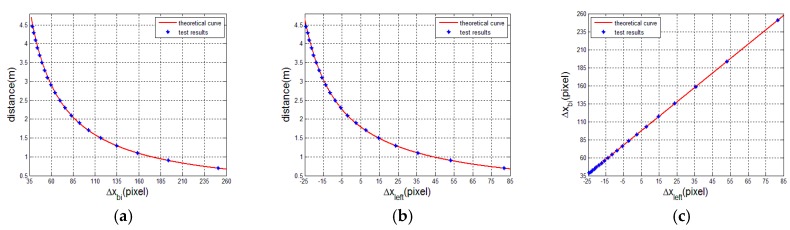
The test results and theoretical curves from Equations (1), (2), and (3). (**a**) The distance curve of displacements $\Delta x_{left}$; (**b**) the distance curve of displacements $\Delta x_{bi}$; (**c**) the transforming curve between displacements $\Delta x_{left}$ and $\Delta x_{bi}$.

In this part, we validate the transforming relationship between the two displacements $\Delta x_{bi}$ and $\Delta x_{left}$ from two matching modes. We capture a set of left and right speckle patterns projected on a standard plane that is perpendicular to the optical axis (Z-axis) of the laser projector and parallel to the left reference speckle pattern plane. The distance information of every speckle pattern is known, and the range is from 0.7 m to 4.46 m. The tested displacements $\Delta x_{bi}$ and $\Delta x_{left}$ for each pattern are estimated and listed in Table 1 and Table 2. In the two tables, the theoretical displacements calculated from Equations (1) and (2) are also listed, where the related camera parameters f=4.387 mm, μ=3.75 μm, the baseline s=74.6 mm, and $d_{r}=2\;m$. From Table 1 and Table 2, it can be found that the error of $\Delta x_{bi}$ is slightly larger than that of $\Delta x_{left}$. The reason is that the baseline of the binocular mode is twice the length of that of the monocular mode. For displacements $\Delta x_{bi}$, from 0.7 to 2.5 m, the error of the test value decreases with the increase in distance. After 2.5 m, the error fluctuation is stable between 0.47 and 0.61. For displacements $\Delta x_{left}$, the error of the test value around the reference distance is at a minimum and the error between the theoretical and test values is no more than one pixel. In Figure 12, we plot the ranging curves calculated from Equations (1) and (2) and the transforming curve from Equation (3) and, additionally, the test displacements are also plotted and represented, shown as blue plots. It intuitively shows that all of the blue plots are basically on the red line.

### 5.2. The Spatial Resolution in X-Y Direction

In this paper, we divide the speckle pattern into blocks (or subsets) to estimate the disparity of every pixel. According to the triangulation principle, the spatial resolution in the X-Y direction is proportional to the size of the divided block and is inversely proportional to distance. The relationship can be expressed as

(8) $$\Delta X\left(Y\right)\propto \frac{m\mu }{f}d,$$

where m represents the block size. In our method, reducing the size of the matching block is performed to improve the spatial resolution of the depth map, but it will reduce the matching accuracy. Therefore, we analyze the error rate of different sizes of blocks at different distances to select the appropriate block size. The two matching modes are performed from 0.7 m to 4.46 m with block sizes of 25 × 25 and 17 × 17, and $\Delta x_{25}$ and $\Delta x_{17}$ represent the estimated displacements, respectively. In the experimental results, we find that, with the larger block size of 25 × 25, there are no mismatching pixels in the two matching modes. Then, when the smaller block size of 17 × 17 is used, for every pixel, if $\left|\Delta x_{25}-\Delta x_{17}\right|>0.25\;\mathrm{pixel}$, we assume that the matching result is an error. Table 3 lists the error rate of the two matching modes with the block size of 17 × 17. The results show that almost no mismatching appears in binocular mode before 3.5 m and the error rate is also very low from 3.7 m to 4.1 m. Therefore, the 17 × 17 block can be selected to perform the DIC algorithm. Moreover, it is obvious that, in monocular mode, the matching results are very fine around the reference distance; in other distances, the mismatching appears—especially at 0.7 m, the mismatching is very serious. In addition, the main calculation is performed in the disparity estimation step (step 5). Although the two matching modes are performed in the present method, we adopt the smaller block without the increase of the calculation amount in comparison to the 25 × 25 block size.

### 5.3. The Analysis and Comparison of Results

**Figure 13:**
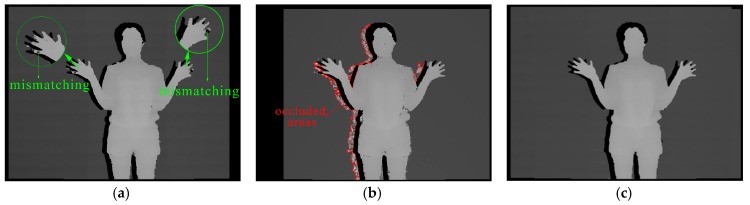
The depth image (**a**) from monocular mode (**b**) from binocular mode (**c**) from the combination of the two matching modes.

Firstly, we employ our method to test people based on our hardware platform and the results are shown in Figure 13. Figure 13a is the output depth image from the monocular mode, with some mismatching happening on the finger part, as shown in the green circle. Figure 13b is the output depth image from the binocular mode, in which the fingers are clearer. However, the occluded areas marked by red curves impact the quality of the whole image. Thus, we correct the occluded areas through Figure 13a to generate the final depth image, as shown in Figure 13c. Comparing Figure 13a with Figure 13b, it is clear that the recognition ability of the monocular mode for tiny objects is lower than that of the binocular mode and mismatching is easier to appear on the similar objects in monocular mode after reducing the block size. Conversely, the noise on the body is slightly greater in Figure 13b. The reason is that the surface of the body is relatively complex. The occlusion also exists on the surface. The black area around the person is the detected shadow area and the final result (Figure 13c) proves that our method can primarily integrate the advantage of Figure 13a,b.

**Figure 14:**
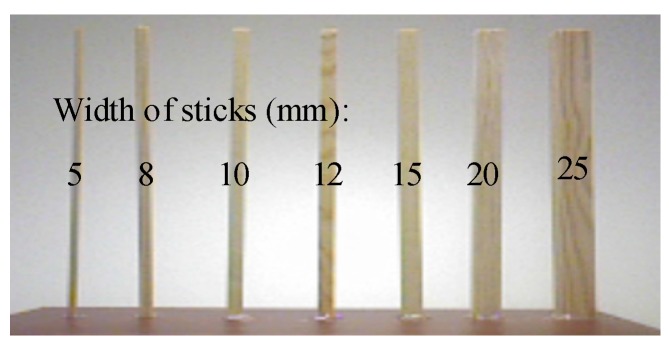
The width of the straight sticks.

**Figure 15:**
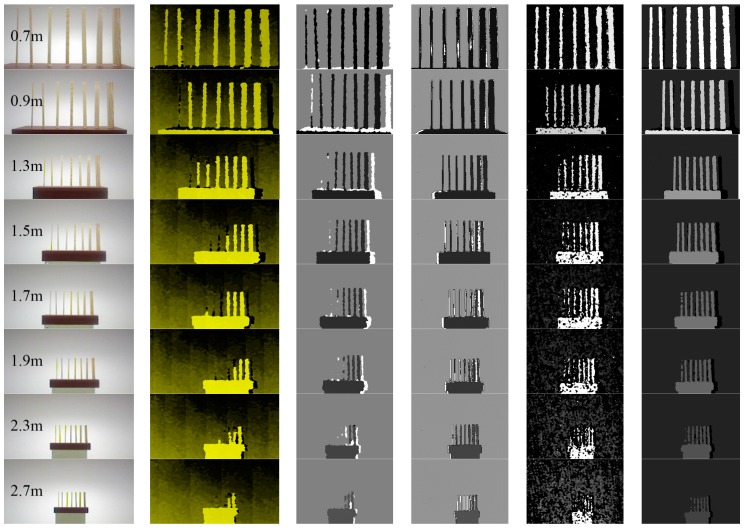

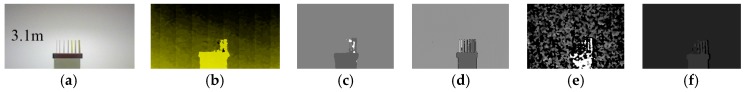
The different widths of the straight sticks (5, 8, 10, 12, 15, 20, and 25 mm from left to right) at different distances. (**a**) Color image; (**b**) Xtion Pro; (**c**) Kinect; (**d**) Kinect 2 (see Appendix A for more details); (**e**) Realsense R200; (**f**) our method.

In order to test the spatial resolution of our system and intuitively compare the results with other similarly devices, such as the Xtion Pro, Kinect, Kinect 2, and Realsense R200, the depth maps on the several straight sticks with known width, as shown in Figure 14, are obtained and shown in Figure 15. The widths of the straight sticks are 5, 8, 10, 12, 15, 20, and 25 mm, from left to right. As shown in Figure 15, the results from our method are always better than that from the Kinect, Xtion Pro and Realsense. For example, it is obvious that the first stick still can be better identified at a testing distance of 1.5 m in Figure 15f, but it only can be identified at 0.7 m in Figure 15b,c,e. At 1.9 m, there are still six sticks in our depth map, but only three sticks in other three maps. Moreover, it is obvious that the noise in Figure 15e is great and the depth map on the flat object always flickers. The reason is that Intel sacrifices the performance of the Realsense in order to reduce the dimensions. The dimensions of the RealSense R200 are only 101.56 mm (length) × 9.55 mm (height) × 3.8 mm (width), which is the smallest depth sensor at present. Compared with the Kinect 2, our results is slightly better before 1.5 m, because the Kinect 2 cannot identify the first stick at 1.5 m. Beyond the testing distance of 1.5 m, our method smooths the depth of the thinner sticks that cannot be tested. However, the Kinect 2 can mark these uncertain areas, although it also cannot obtain the depth value of these objects, such as the location of the first stick from 1.5 m to 3.1 m. Although there are some problems in our method, the results from Figure 13 and Figure 15 still prove that the present method not only improves the spatial resolution of the depth image, but also ensures the quality of depth image.

In Figure 15, we simplify the tested scene and objects, measuring the spatial resolution of different devices under an ideal circumstance. The results are optimal to a large extent. In practical applications, the scenes and structure of objects are complicated, and the tested results will be affected. Therefore, in the following, we presented more experimental results and discussed them.

**Figure 16:**
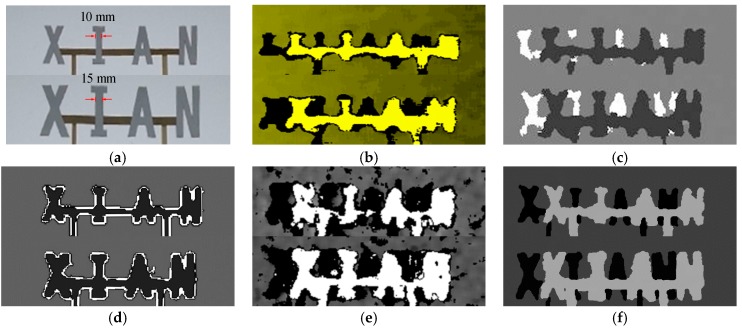
The test results on some capital letters. (**a**) Color image; (**b**) Xtion Pro; (**c**) Kinect; (**d**) Kinect 2 (see Appendix A for more details); (**e**) Realsense R200; (**f**) our method.

As shown in Figure 16, we tried our system on some capital letters. The strokes are 10 mm (the upper line) and 15 mm (the lower line), respectively, which have been labeled in Figure 16a. The testing distance is about 1.15 m. In Figure 14 and Figure 15, sticks with the width of 10 mm and 15 mm are the third and fifth ones, respectively. It can be concluded that the two sticks can be tested by these depth sensing devices at 1.15 m. The test results in Figure 16 are consistent with this conclusion. All devices can detect that there are some objects at this position. However, it is different to accurately recognize which letters they are. For example, for the letter “N,” in Figure 16b,c,e, the test results in the upper line are poor. In the lower line, the results in Figure 16b,c slightly improve, but they look more like the letter “H.” Figure 16d,f show an improvement over the other three, but there are also problems in the test results. The holes in the letter “A” are not detected by our system or Kinect 2.

**Figure 17:**
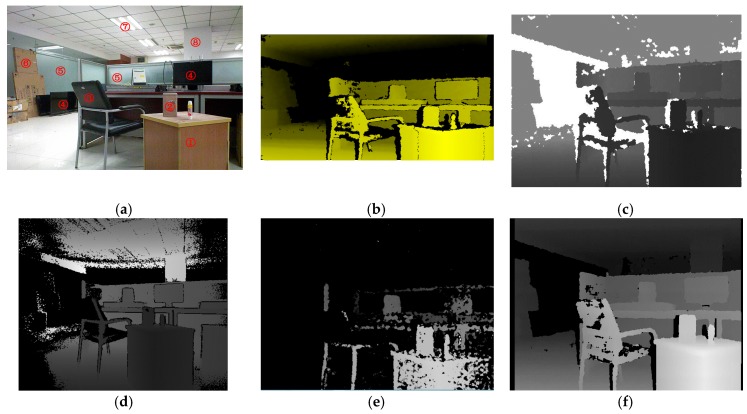
The test results in the complex scene with furniture. (**a**) Color image; (**b**) Xtion Pro; (**c**) Kinect; (**d**) Kinect 2; (**e**) Realsense R200; (**f**) our method.

At last, we performed an experiment in a complex scene with furniture and present the results here in Figure 17. Table 4 shows the working mode and performance features of every device. The Xtion and Kinect employ the same chip (Primesense P1080), and the performance features are almost the same, so we do not list them in this table. Firstly, from Figure 17, we can see that the depth capture distance of our system and Kinect 2 is obviously an improvement over the other devices. Only Object ⑧ can be tested in Figure 17d,f. Secondly, the Kinect 2 adopts ToF technology, while other devices are all based on structured light. Object ⑥ can be tested in Figure 17b,c,f. However, Kinect 2 cannot be detected it in its range limit. Therefore, we consider that sometimes the structured-light technology is better than the ToF technology. Finally, the optical phenomena will affect the measurement results. In Figure 17d, there are some noises on Object ②, which is a mirror reflection object. The surface of Object ③ absorbs the light, which significantly affects the performance of the depth measurement based on active vision.

## 6. Conclusions

In this paper, an FPGA hardware design method for a depth sensing system based on active structured light is presented to improve the spatial resolution of the depth image with two infrared cameras. Firstly, the infrared cameras, based on narrow band-pass filtering, cooperate with the infrared laser to capture the clearer speckle pattern, which is projected by laser and used to mark the space. Secondly, we improved the spatial resolution in the X-Y direction by reducing the size of the matching block, but the smaller size reduces the matching strain precision. Thus, the two matching modes are combined to obtain more precise data. Moreover, the method solves the occlusion problem existing in traditional binocular stereo systems. Thirdly, we introduce the full pipeline architecture of the depth sensing method and verified it on an FPGA platform. However, if our system is to be widely adopted in the future, some optimization is needed to limit hardware resources and power consumption, and more and various scenes are also needed to verify the system; this will be the focus of our future work.

## Figures and Tables

**Table 1:** A list of the displacements $\Delta x_{bi}$ at different distance.

Distance(m)	Theory Value	Test Value	Distance(m)	Theory Value	Test Value
0.7	250.06	250.79 ± 0.94	2.7	64.67	64.21 ± 0.51
0.9	193.08	193.66 ± 0.75	2.9	60.08	59.60 ± 0.59
1.1	157.96	158.58 ± 0.71	3.1	56.21	55.71 ± 0.57
1.3	134.26	135.05 ± 0.74	3.3	52.88	52.35 ± 0.63
1.5	116.36	116.83 ± 0.65	3.5	49.91	49.33 ± 0.58
1.7	102.55	102.73 ± 0.61	3.7	47.19	46.62 ± 0.58
1.9	92.06	92.06 ± 0.53	3.9	44.80	44.24 ± 0.61
2.1	83.12	83.15 ± 0.56	4.1	42.61	42.02 ± 0.47
2.3	75.76	75.70 ± 0.53	4.3	40.64	40.04 ± 0.50
2.5	70.07	69.99 ± 0.50	4.46	39.11	38.45 ± 0.58

**Table 2:** A list of the displacements $\Delta x_{left}$ at different distance.

Distance(m)	Theory Value	Test Value	Distance(m)	Theory Value	Test Value
0.7	81.40	81.79 ± 0.67	2.7	−11.30	−11.37 ± 0.80
0.9	52.90	53.20 ± 0.59	2.9	−13.59	−13.75 ± 0.58
1.1	35.34	35.64 ± 0.58	3.1	−15.53	−15.67 ± 0.43
1.3	23.50	23.87 ± 0.58	3.3	−17.20	−17.36 ± 0.43
1.5	14.55	14.75 ± 0.47	3.5	−18.68	−18.89 ± 0.42
1.7	7.64	7.73 ± 0.32	3.7	−20.04	−20.22 ± 0.37
1.9	2.39	2.41 ± 0.28	3.9	−21.24	−21.37 ± 0.44
2.1	−2.08	−2.09 ± 0.30	4.1	−22.33	−22.49 ± 0.50
2.3	−5.76	−5.69 ± 0.51	4.3	−23.32	−23.45 ± 0.45
2.5	−8.60	−8.72 ± 0.68	4.46	−24.08	−24.28 ± 0.50

**Table 3:** The error rate (‰) of the two matching modes at different distance.

Distance (m)	Binocular Mode	Monocular Mode	Distance (m)	Binocular Mode	Monocular Mode
0.7	0	0.48	2.7	0	0.03
0.9	0	0.22	2.9	0	0.03
1.1	0	0.18	3.1	0	0.04
1.3	0	0.06	3.3	0	0.03
1.5	0	0	3.5	0	0.08
1.7	0	0	3.7	0.01	0.07
1.9	0	0	3.9	0.01	0.06
2.1	0	0	4.1	0.03	0.06
2.3	0	0	4.3	0.09	0.06
2.5	0	0	4.46	0.20	0.09

**Table 4:** The performance comparison.

Item	Kinect	Kinect 2	Realsense R200	Our Method
Working mode	Structured light	ToF	Structured light	Structured light
Range limit	0.8~3.5 m	0.5~4.5 m	0.4~2.8 m	0.8~4.5 m
Framerate	30 fps	30 fps	Up to 60 fps	Up to 60 fps
Image resolution	640 × 480	512 × 424	Up to 640 × 480	Up to 1280 × 960
Bits of depth image	10bits	unpublished	12bits	12bits
Vertical Field	43°	60°	46° ± 5°	43°
Horizontal Field	57°	70°	59° ± 5°	58°
Output interface	USB 2.0	USB 3.0	USB 3.0	USB 3.0

**Figure A1:**
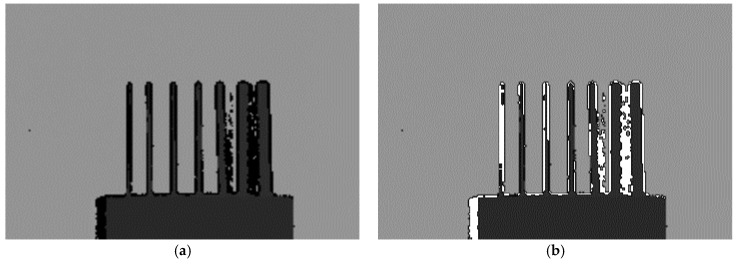
Depth images at distance 1.5 m from Kinect 2: (**a**) original depth image; (**b**) revised depth image.

## Appendix A

In the depth images from the Kinect 2 in Figure 15d, the uncertain areas in the depth images are marked by dark pixels. This makes the depth of the measured sticks difficult to be distinguished from the uncertain area. Therefore, we made a small change in Figure 15d, as shown in Figure A1b; the uncertain areas are marked by white pixels.
